# A Bottom-Up Multi-Feature Fusion Algorithm for Individual Tree Segmentation in Dense Rubber Tree Plantations Using Unmanned Aerial Vehicle–Light Detecting and Ranging

**DOI:** 10.3390/plants14111640

**Published:** 2025-05-27

**Authors:** Zhipeng Zeng, Junpeng Miao, Xiao Huang, Peng Chen, Ping Zhou, Junxiang Tan, Xiangjun Wang

**Affiliations:** 1 Rubber Research Institute, Chinese Academy of Tropical Agricultural Sciences, Haikou 571101, China; 18280060078@163.com (Z.Z.); 13976786119@163.com (X.H.); 2 College of Earth Sciences, Chengdu University of Technology, Chengdu 610059, China; miaojunpeng@stu.cdut.edu.cn (J.M.); chenpeng1@stu.cdut.edu.cn (P.C.); 3 The 4th Geological Brigade of Sichuan, Chengdu 611130, China; merci0929@163.com

**Keywords:** individual tree segmentation, LiDAR point clouds, rubber plantations, bottom-up segmentation, multi-feature fusion, precision forestry

## Abstract

Accurate individual tree segmentation (ITS) in dense rubber plantations is a challenging task due to overlapping canopies, indistinct tree apexes, and intricate branch structures. To address these challenges, we propose a bottom-up, multi-feature fusion method for segmenting rubber trees using UAV-LiDAR point clouds. Our approach first involves performing a trunk extraction based on branch-point density variations and neighborhood directional features, which allows for the precise separation of trunks from overlapping canopies. Next, we introduce a multi-feature fusion strategy that replaces single-threshold constraints, integrating geometric, directional, and density attributes to classify core canopy points, boundary points, and overlapping regions. Disputed points are then iteratively assigned to adjacent trees based on neighborhood growth angle consistency, enhancing the robustness of the segmentation. Experiments conducted in rubber plantations with varying canopy closure (low, medium, and high) show accuracies of 0.97, 0.98, and 0.95. Additionally, the crown width and canopy projection area derived from the segmented individual tree point clouds are highly consistent with ground truth data, with R^2^ values exceeding 0.98 and 0.97, respectively. The proposed method provides a reliable foundation for 3D tree modeling and biomass estimation in structurally complex plantations, advancing precision forestry and ecosystem assessment by overcoming the critical limitations of existing ITS approaches in high-closure tropical agroforests.

## 1. Introduction

Rubber plantations play a significant role as artificial forest ecosystems in tropical regions. The extraction and mapping of structural parameters from these plantations are crucial for various applications, including ecosystem assessment [1], tree species cultivation and breeding [2], refined management [3], and carbon sink quantification [4]. Traditional methods for acquiring structural data primarily rely on field measurements, which are often time-consuming and labor-intensive [5]. Recently, Light Detection and Ranging (LiDAR) technology has garnered extensive attention due to its ability to rapidly and accurately capture the vertical structure information of forests [6]. In particular, employing low-altitude unmanned aerial vehicles (UAVs) can generate high-density point cloud data. This approach is characterized by its strong operability, high geometric resolution, and detailed delineation of tree structures [7]. A key challenge lies in accurately segmenting individual tree point clouds from large-scale data, which is essential for extracting individual tree structural parameters and constructing three-dimensional tree models, as well as for precise biomass estimation.

Currently, methods for the individual tree segmentation (ITS) of LiDAR point clouds can be broadly classified into two categories: segmentation based on projected images [8] and direct segmentation based on point clouds [9] and emerging deep learning approaches [10,11,12,13]. Segmentation based on projected images generates raster images through the interpolation of the Canopy Height Model (CHM), identifies tree top points using local extremum algorithms, and employs clustering algorithms to estimate the canopy boundaries for individual trees. Koch et al. [14] proposed an ITS approach utilizing the water-filling algorithm, which starts from local maxima and expands into neighboring areas of lower height values. In coniferous forests with lower canopy closure, this algorithm corresponds well with the actual canopy boundaries; however, it may lead to over-segmentation in regions where canopies overlap [15]. Chen et al. [16] introduced a marker-controlled watershed algorithm that segments trees based on specific marked positions rather than local maxima. This method has shown effective results in coniferous forests, but it struggles with dense deciduous forests where canopy shapes are complex and overlapping. Jing et al. [17] presented an ITS method that integrates multi-scale filtering algorithms. By initially smoothing the grayscale image using a Gaussian filter, followed by segmentation with the watershed algorithm, this approach effectively reduces over-segmentation in cases with similar canopy shapes. However, multi-scale filtering can blur canopy boundaries, leading to rougher segmentation that does not align precisely with the actual canopy edges. These ITS methods, which rely on the identification of tree top points from the CHM, work well in coniferous forests with clear tree tops but struggle in broadleaf forests with less distinguishable tree tops [18].

Direct ITS methods based on point clouds are categorized into two types: top-down and bottom-up. Top-down methods use tree top points as seed points to detect individual trees. Reitberger et al. [19] applied a graph-theory-based Normalized Cut (Ncut) method for ITS, using the positions of tree tops and trunks as prior knowledge. This method addresses the issue faced by segmentation techniques based on projected images, which have difficulty detecting small trees in the middle and lower layers. However, the Ncut method becomes computationally demanding when applied to high-density point cloud data, reducing its efficiency. Li et al. [20] employed a distance threshold for detecting relative distances between trees, achieving excellent segmentation in mixed coniferous forests. Ayrey et al. [21] introduced a layer stacking algorithm that slices the normalized point cloud into layers and performs top-down ITS based on these slices. This method reduces data loss in the creation of raster surfaces and models from the CHM and improves segmentation accuracy by using adaptive thresholds and additional classification rules. Nonetheless, this approach still relies on the accurate identification of tree top positions and is limited in stands with poorly defined tree tops.

Bottom-up ITS methods begin with tree trunks and use trunk points as seed points, which enables the more accurate association of tree crowns with their corresponding trunks. Lu et al. [22] and Reitberger et al. [23] both achieved favorable results in segmenting tree trunks by leveraging horizontal distances among point cloud data. Lu et al. [22] employed a three-dimensional line refinement approach based on Random Sample Consensus (RANSAC) to detect tree trunks, while Reitberger et al. [23] filtered out small branches and leaf points based on intensity values before detecting tree trunks. Torchta et al. [24] initially divided trees into horizontal slices, extracted eligible clusters based on point distance, and merged clusters with the fewest points. The final step involved determining the angle and distance of the cluster center to segment individual trees. However, this method struggled in dense forest environments, as missing data can significantly impact segmentation performance. Shendryk et al. [25] proposed a random walk algorithm for segmenting individual tree point clouds in eucalyptus forests using full-waveform LiDAR data. This approach calculates the path probability between points and tree trunks for segmentation, but it faces difficulties distinguishing overlapping tree crowns. Jaskierniak et al. [26] introduced a bottom-up ITS method for eucalyptus mixed forests, which involves rasterizing the tree trunk point cloud, eliminating low vegetation, and applying an improved watershed algorithm for hierarchical clustering. This approach has shown improvements in handling forests with complex structures, but segmenting tree crown edges in areas with severe branch overlap remains a challenge.

In recent years, deep learning has emerged as a powerful tool in the field of single tree segmentation. Xiang et al. [27] developed the ForAINet framework, which can perform semantic, instance, and component segmentation on high-density airborne LiDAR point clouds, achieving high F-scores and mean IoU values in segmenting individual trees and tree components. Wielgosz et al. [28] proposed the SegmentAnyTree model, which is sensor- and platform-agnostic and can effectively segment trees from various laser scanning data. These deep-learning-based methods have shown great potential in improving the accuracy and efficiency of single tree segmentation compared to traditional methods [29,30]. However, deep-learning-based methods also have some limitations. For example, they usually require a large amount of labeled data for training, and the quality of the training data have a great impact on the performance of the model. In addition, these models are often complex in structure, resulting in high computational costs and long training times. Moreover, when dealing with complex forest structures or low-density point cloud data, the segmentation accuracy of deep-learning-based methods may also be affected.

In China, the prevalent planting patterns for rubber plantations typically include rectangular planting (with tree spacing of 3–4 m and row spacing of 6–7 m) or street-style planting (with tree spacing of 1.5–3 m and row spacing of 8–12 m). Mature rubber plantations often exhibit high canopy closure, leading to intense competition among branches and leaves, and resulting in significant canopy overlap. Despite the numerous ITS methods proposed in previous studies, accurately segmenting tree canopy edges in rubber plantations remains a considerable challenge due to the interwoven canopies. Therefore, there is an urgent need to develop a robust and versatile ITS method specifically suited for the precise segmentation of rubber tree canopies. Such a method would provide an essential foundation for accurate individual tree structural parameter extraction, three-dimensional model construction, and biomass inversion.

To address the challenges of indistinct canopy vertices, severe branch overlap, and difficulty in precisely segmenting individual tree point clouds in rubber plantations, this paper proposes a bottom-up growing ITS method for LiDAR point clouds from rubber plantations. The key contributions of this work are as follows:

(1) Density–angularity fused trunk extraction: A main trunk extraction method based on branch point identification is introduced. By analyzing density and angle variations at branch points combined with neighborhood point density thresholds and main direction features, this method precisely segments the main trunk and canopy. The method processes individual tree point cloud data from bottom to top, improving the accuracy and efficiency of tree structure analysis.

(2) Multi-feature constrained crown delineation: Multi-feature fusion is employed, replacing the single threshold limitation for ITS. By constructing multiple features for constraints, this method achieves the precise segmentation of canopy core points, boundary points, and disputed points in overlapping branches. The discrimination of disputed points, based on the consistency of growth direction angles within the neighborhood range, ensures the accurate attribution of points in overlapping branches, thus enhancing segmentation accuracy and noise resistance. This method demonstrates strong segmentation results for canopies with low, medium, and high closure levels.

## 2. Results

### 2.1. Parameter Sensitivity Analysis

**Figure 1:**
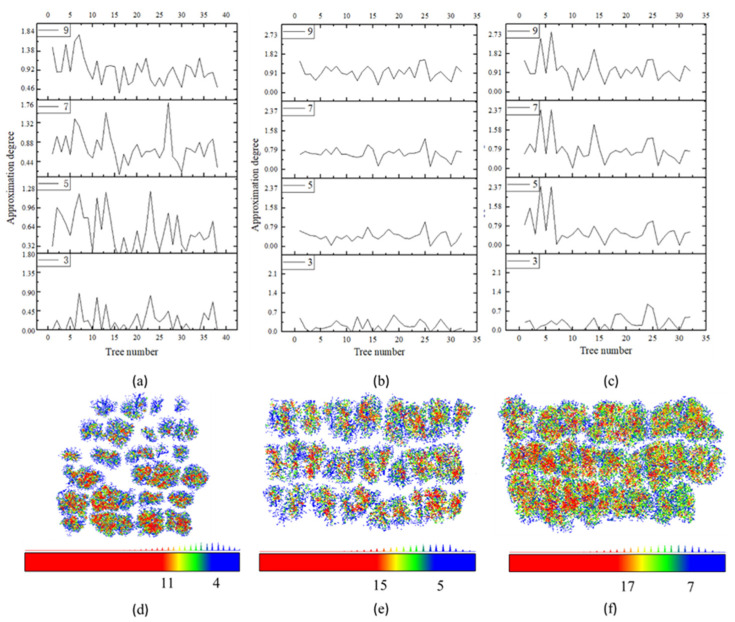
Sensitivity analysis of distance and density parameters for identifying branch points and classifying core and boundary skeleton points. (**a**–**c**) Results of branch point identification based on varying density thresholds *ρ*_1_ in plots A, B, and C, respectively. (**d**–**f**) Results of core and boundary skeleton point classification using set density thresholds *ρ*_2_ and *ρ*_3_ in plots A, B, and C, respectively. Core points are depicted in red, boundary points in blue, and non-skeleton points in green.

The density of skeleton points for both the trunk and canopy exhibits significant variation, yet the main directional orientation of the skeleton points from bottom to top remains relatively consistent. When the density variation exceeds the preset value *ρ*_1_, and the angle between the direction vector of the upper- and lower-layer skeleton points and the primary direction does not surpass the threshold angle *θ*th, the point is categorized as a branch point. To quantify the degree of approximation between the algorithm-generated branch points and the manually extracted branch points, the Euclidean distance between them is calculated. A sensitivity analysis of the density threshold *ρ*_1_ is then performed. As illustrated in Figure 1a–c, when *ρ*_1_ is set to 3, the algorithm-generated branch points are found to be more closely aligned with the manually extracted branch points.

For the crown width in both the row and column directions of the rubber tree, *T_HD1_* is set to approximate the minimum crown radius of the sample plot, while *T_HD2_* corresponds to the maximum crown radius. These two distance parameters are employed to distinguish core points from non-core points. The values for *T_HD1_* are 1.5 m, 1.5 m, and 1.2 m in plots A, B, and C, respectively. Similarly, the values for *T_HD2_* are 5 m, 3 m, and 3 m in plots A, B, and C, respectively.

The parameter *ρ_2_* is used as a secondary criterion to differentiate core points from non-core points, with core points having a higher local density than non-core points. By conducting a neighborhood search within a radius of 0.4 m around the skeleton points in the sample plot, skeleton points with varying local densities are represented in different colors—core points in red and boundary points in blue, as shown in Figure 1d–f. Core points fall within the local density range of *ρ* ≥ *ρ*_3_ and the horizontal distance of branch points (0, *T_HD1_*), while boundary points are within the range of *ρ* < *ρ*_3_ and the horizontal distance of branch points (*T_HD1_*, *T_HD2_*). Skeleton points with *ρ* < *ρ*_2_ and within the horizontal distance range of branch points (*T_HD1_*, *T_HD2_*) are designated as controversial points.

The corresponding density thresholds *ρ*_2_ and *ρ*_3_ used to segment core and boundary points are established based on the local density of skeleton points in different sample plots, each exhibiting varying canopy closure levels. For plot A, with low canopy closure, *ρ*_2_ is set to 4, and *ρ*_3_ is set to 11; for plot B, with medium canopy closure, *ρ*_2_ is set to 5, and *ρ*_3_ is set to 15; for plot C, with high canopy closure, *ρ*_2_ is set to 7, and *ρ*_3_ is set to 17. An angle *θ* is then defined between the existing classified skeleton points and the unclassified skeleton points (controversial points) based on the growth characteristics of the branches. This angle aligns with the growth trend observed between the branch skeleton points or between the branch and leaf skeleton points.

### 2.2. Comparison with Existing Methods

**Figure 2:**
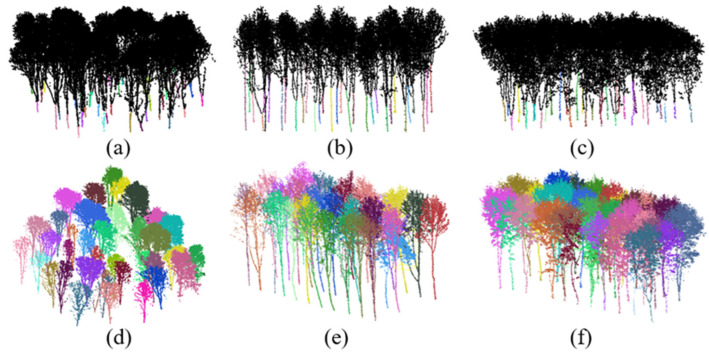
The results of trunk skeleton extraction and individual tree segmentation from the point clouds of the three sample plots. (**a**–**c**) The colorized points represent the extracted trunk skeletons in sample plot A, B, and C, respectively; (**d**–**f**) the segmented point clouds of individual trees rendered in different colors in sample plot A, B, and C, respectively.

The algorithm discussed previously was applied to rubber tree plantation stands with low (Plot A), medium (Plot B), and high (Plot C) canopy closure, for the purposes of main trunk extraction and canopy segmentation. Figure 2 presents the color-coded point cloud results of the extracted trunks and the segmented individual trees in the three plots. The methodology demonstrated consistent precision in trunk identification, generating discrete tree point clouds that preserved intact morphological structures, regardless of variations in canopy closure.

To validate the accuracy of our proposed ITS algorithm, the quantities of correctly segmented, under-segmented, and over-segmented individual trees were calculated using a point percentage criterion for each of the three sample plots. The results, as shown in Table 1, indicate that our algorithm achieved high segmentation accuracies in all three plots, with minimal over-segmentation. Specifically, the overall accuracies were 97% for Plot A and 98% for Plot B. Although Plot A exhibited a lower canopy closure than Plot B, data gaps in its tree trunk point cloud led to the premature termination of the algorithm at trunk breaks, resulting in a slightly lower overall accuracy compared to Plot B. While Plot B also showed some branch overlap among the rubber trees, its higher data integrity led to improved algorithm recognition performance. Plot C, which had the highest canopy closure, demonstrated a slightly lower overall accuracy of 95%. This lower accuracy was primarily due to significant branch overlap, which posed challenges for accurately identifying canopy boundaries across all rubber trees in the plot. Despite this, the segmentation accuracy in Plot C was only 0.02 lower than that in Plot A, the plot with the lowest canopy closure.

To further validate the advantage of the algorithm presented in this paper, a comparative analysis was conducted between the ITS results of our algorithm and those of existing methods, including the hierarchical DBSCAN algorithm [31], point cloud segmentation (PCS) [20], the ForAINet algorithm [27], the layer stacking algorithm [32], the K-means algorithm [33], and the watershed algorithm [34]. This study employed the ForAINet network to train a model using single-tree point cloud data from rubber plantations. Once the model was trained, it was applied to predict single-tree point clouds in three subregions—A, B, and C—of the experimental area, thereby achieving the single-tree segmentation of point clouds based on deep learning. These six algorithms are commonly employed for the ITS of UAV-LiDAR point clouds. Among these, the hierarchical DBSCAN algorithm was also the method used for extracting tree skeleton points in this paper.

**Figure 3:**
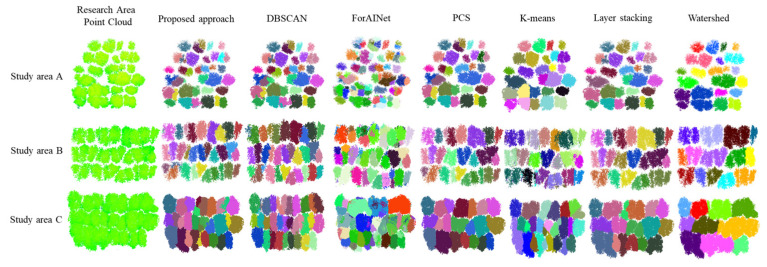
Tree segmentation results of different algorithms in three sample plots.

As shown in Figure 3, the segmentation accuracies of the other five algorithms decline significantly as the canopy density of the rubber forest increases. In contrast, the performance of our proposed approach remains highly stable across sample plots with varying canopy closure. The results in Table 1 further highlight that the ITS accuracy of our algorithm is significantly superior to the other five algorithms in all three plots with differing canopy closures. Among the comparative methods, the hierarchical DBSCAN algorithm achieved the second-highest segmentation accuracy. However, its performance declined sharply with increasing canopy density, indicating that the hierarchical DBSCAN algorithm struggled with segmenting complex and overlapping tree crowns. This is because the hierarchical DBSCAN algorithm is based on global point cloud density, whereas the region growing algorithm segments are only based on local neighborhood information. When handling complex tree crown point clouds, the region growing algorithm is more prone to errors, resulting in lower segmentation accuracy compared to the hierarchical DBSCAN algorithm.

The K-means, layer stacking, and watershed algorithms performed the worst across all three sample plots. This may be due to the fact that these algorithms all use a top-down segmentation approach. Given the significant overlap of rubber tree crowns and the indistinct tree top points, these algorithms tend to suffer from over-segmentation and under-segmentation. In contrast, our proposed algorithm begins by acquiring the lowest point of the tree trunk as the initial seed point, working from the bottom to the top. This approach bypasses the difficulties of obtaining the initial seed point in complex tree crown structures. Additionally, by using thresholds such as angle, distance, and density between skeleton points as constraints, the algorithm effectively resolves the limitations of single-threshold segmentation, enabling it to handle complex point clouds more accurately.

### 2.3. Accuracy Assessment of Individual Tree Parameter Extraction

The accuracy of ITS, particularly in crown segmentation, directly impacts the precision of extracted individual tree morphological parameters such as crown width and canopy projection area. To evaluate the performance of our algorithm, we used point cloud data segmented by this method to calculate the individual tree crown width and canopy projection area of rubber trees. These values were then compared to those obtained from manually segmented point clouds, which served as ground truth data.

**Figure 4:**
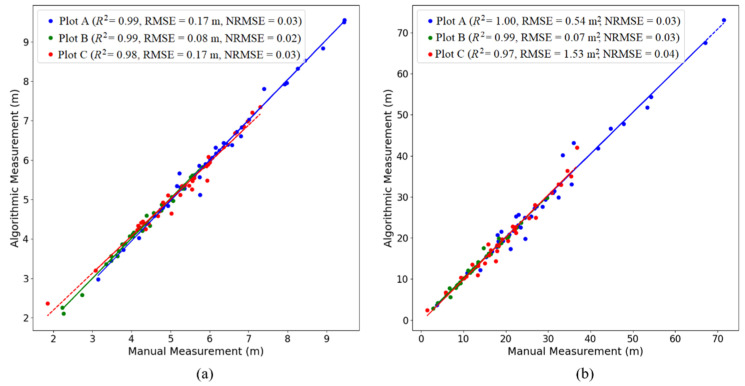
The fitting line plots of the extracted and measured values of the canopy parameters of the three sample plots. (**a**): Crown width; (**b**): canopy projection area.

As shown in Figure 4, in plots A, B, and C with progressively increasing canopy closure, the R^2^ values for crown width were 0.99, 0.98, and 0.97, respectively, and the *R*^2^ values for canopy projection area were 1.00, 0.99, and 0.97, respectively. These high *R*^2^ values indicate a strong agreement between the crown width and canopy projection area derived from algorithm-segmented point clouds and the manually segmented results, effectively validating the accuracy of our ITS algorithm. Therefore, the point clouds segmented by our algorithm are capable of accurately extracting individual tree parameters. However, it is important to note that as the canopy closure of rubber plantations increases, the accuracy of ITS may decrease slightly, potentially affecting the precision of individual tree parameter extraction.

## 3. Discussion

The bottom-up ITS algorithm presented in this paper is sensitive to the influence of understory vegetation, particularly understory shrubs. Rubber plantations are artificial economic forests, with their stand structure comprising the tree layer, shrub layer, and herb layer. Unlike other tropical forests, rubber plantations have a single tree layer, which consists entirely of rubber trees distributed uniformly. The shrub layer contains relatively few plants, while the herb layer has a higher density of vegetation [35]. Consequently, during the data preprocessing stage, the feature identification of understory vegetation point clouds can be performed, or deep learning algorithms can be employed for the semantic segmentation of rubber trees and understory vegetation. By eliminating the understory vegetation point clouds from the original data, the influence of understory vegetation on the ITS of rubber trees can be minimized.

The main trunk of rubber trees has a relatively simple morphological structure, and a uniform point cloud density threshold can effectively segment the trees. However, the morphological structure of the tree crown is more complex, with significant variations in point cloud density and branching angles among individual tree crowns. A uniform density and angle threshold are ineffective for segmenting the tree crown. In this paper, the point cloud density and angle thresholds are manually adjusted based on experience to achieve the effective segmentation of the tree crown. Wu et al. [36] utilized Edge Convolution [37] to encode the local neighborhood of points as a directed graph, followed by employing a multi-layer perceptron to abstract points and adjacent edges into edge feature vectors. Max-pooling was then used to aggregate these edge feature vectors into local spatial features of points. This method could be adapted to extract the local density and angle features of rubber tree crown point clouds and develop an adaptive threshold segmentation process [38,39] for individual tree point clouds, which would enhance the accuracy and efficiency of the algorithm presented in this paper.

In mature rubber plantations, rubber trees grow vigorously, and there is a significant overlap of branches and trunks among different individual trees. As a result, the precise segmentation of tree crowns presents a challenge in the point cloud segmentation of individual rubber trees. The algorithm proposed in this paper utilizes UAV-LiDAR point clouds, adopts a top-down skeleton growth approach, and integrates point cloud distance, density, and angle characteristics to accurately segment individual rubber trees and their crowns. The algorithm in this paper is a hard segmentation method. Dai et al. [40] proposed a soft segmentation algorithm, which can automatically segment complex and severely overlapping crown regions and reconstruct the crown surface, improving crown segmentation accuracy by 90% compared to hard segmentation. Additionally, by using multi-sensor data fusion technology, combining airborne laser scanning (ALS) with terrestrial laser scanning (TLS) and multispectral or hyperspectral data, the accuracy of individual tree crown segmentation in dense tropical forests can be enhanced [41,42]. Future studies can employ multi-sensor fusion data and soft segmentation methods to handle complex and overlapping crown point clouds, further improving the segmentation accuracy of individual rubber trees, particularly for crown point clouds.

ITS serves as a foundational methodology for quantifying rubber tree architectural traits, where vertically distributed parameters (such as tree height and under-branch height), angular features (such as branching angle), and crown characteristics (such as crown width, volume, and foliage density) collectively determine wood volume and wind resistance. While trunk-related metrics tend to remain stable across segmentation variations, crown parameter extraction is more sensitive to point cloud processing accuracy due to the topological complexity of the crown. This differential error propagation necessitates stratification in predictive modeling, requiring the error-weighted integration of crown-derived and trunk-based indicators when constructing composite evaluation indices for silvicultural applications, such as timber production and wind resistance assessments.

As noted above, ITS plays a significant role in rubber plantation research, including aboveground biomass estimation. Since rubber plantations are major carbon reservoirs, accurately estimating their biomass contributes to understanding the carbon cycle in terrestrial ecosystems [43]. Moreover, obtaining precise biomass values for rubber plantations enables the formulation of appropriate management strategies [44]. Although several methods for estimating forest aboveground biomass based on ITS have been developed [45,46,47], some ITS methods based on UAV-LiDAR point clouds may lead to the overestimation or underestimation of the total tree count in the study area. Therefore, when evaluating forest biomass based on ITS, it is essential to account for potential overestimation or underestimation in light of the performance of the relevant methods.

## 4. Materials and Methods

### 4.1. Data Acquisition

**Figure 5:**
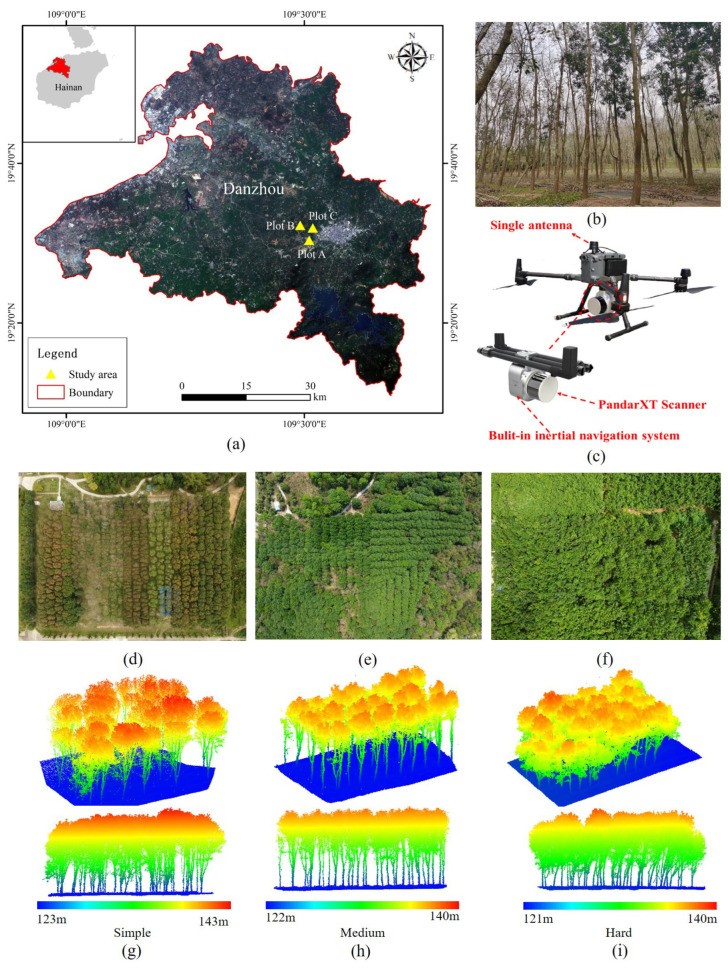
Aerial photographs and LiDAR point clouds of three sample plots from the rubber tree experimental base in Danzhou City, located in northwest Hainan Province, China. (**a**) Map showing the study area location; (**b**) rubber plantation landscape; (**c**) CBI-Lite LiDAR Measurement System; (**d**–**f**) orthorectified images of the study areas; (**g**–**i**) UAV-LiDAR point clouds displaying varying levels of canopy closure: low, medium, and high.

The data were collected at the rubber tree experimental base in Danzhou City, located in the northwest of Hainan Province (as shown in Figure 5). Danzhou is situated between 19°11′ and 19°52′ north latitude and 108°56′ and 109°46′ east longitude. It is the largest rubber production base on Hainan Island, known for its diverse range of rubber tree varieties and abundant rubber resources. The city has a tropical monsoon climate, with an average annual sunshine duration exceeding 2000 h. The average annual temperature is 23.2 °C, and the average annual rainfall is 1815 mm.

To evaluate the performance of the single-tree segmentation algorithm presented in this paper, rubber tree point cloud data were acquired from three sample plots—A, B, and C—representing low, medium, and high canopy closure levels, respectively. The locations of these plots are shown in Figure 5a, and the data for each plot are provided in Table 2.

The experiment utilized a DJI M300RTK unmanned aerial vehicle (UAV) (DJI Innovation Technology Co., Ltd., Shenzhen, China) equipped with a CBI-Lite LiDAR measurement system (Chengdu Alundar Technology Co., Ltd., Chengdu, China) (as illustrated in Figure 5b) to collect data from the rubber forest during the leaf-falling period. Data acquisition took place on 5 March 2023. The LiDAR point clouds obtained are shown in Figure 5g–i. The UAV flew at an altitude of 80 m, maintaining a distance of 40–50 m from the top of the tree canopy, with an average speed of 6 m/s. The flight path had a lateral overlap of 60%, and the spacing between flight paths was 10 m.

The CBI-Lite LiDAR system was equipped with a PandarXT-customized LiDAR sensor, which operates with a laser emission wavelength of 905 nm, a scanning frequency of 10 Hz, and a 360° angular range. This system can generate approximately 1.92 million points per second, with an effective detection range of up to 120 m.

### 4.2. Data Preprocessing

The raw LiDAR data collected from rubber plantations contains both ground points and noise information. To obtain clean rubber tree point clouds, essential preprocessing steps are required, including point cloud denoising and ground filtering. This study implements outlier detection [48] for denoising the point cloud data in CloudCompare (v2.12.4), followed by the application of the Cloth Simulation Filter (CSF) algorithm [49] through the CSF plugin in CloudCompare to differentiate between ground and non-ground points (parameters: resolution = 0.5 m, rigidness = 3). Furthermore, to accurately represent tree heights, vegetation points undergo elevation normalization within the same software environment. This process involves generating a Digital Elevation Model (DEM) through the Inverse Distance Weight (IDW) interpolation of ground points using CloudCompare’s Rasterize tool (power = 2, search radius = 1.5 m), and then calculating the difference between vegetation point elevations and the DEM to produce the normalized point cloud [50].

### 4.3. Bottom-Up Multi-Feature Fusion Algorithm for ITS

**Figure 6:**
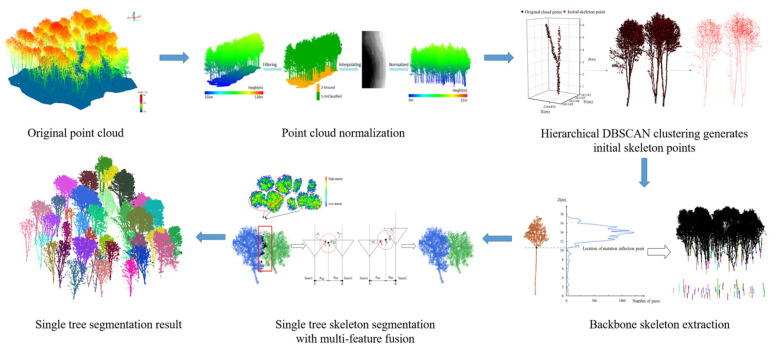
Workflow of the proposed ITS method.

The proposed bottom-up multi-feature fusion algorithm for Intelligent Tree Segmentation (ITS) consists of three primary stages: (1) generation of initial skeleton points, (2) extraction of the main trunk skeleton of individual trees, and (3) ITS based on multi-feature fusion. In the first stage, the point cloud of the sample site is vertically stratified, and the centroids of the clusters are determined through bottom-up clustering. These centroids act as the initial skeleton points for the rubber plantation. The initial skeleton points are representative of the actual tree morphology, and their generation helps mitigate the effects of uneven dataset density distribution on clustering outcomes. Next, branch points are identified by analyzing the consistency of the main trunk points’ directions and the density contrast between the trunk and the canopy. These branch points serve as seed points for segmenting the main trunk and the canopy, preventing the mis-segmentation that could arise from using a uniform feature threshold. Finally, to improve ITS accuracy in scenarios with complex or overlapping canopies, multi-feature fusion is employed to process the skeleton points across different canopy regions. The overall process is illustrated in Figure 6.

#### 4.3.1. Generation of Initial Skeleton Points

The bottom-up ITS, which utilizes point-by-point analysis and calculation, demonstrates relatively low processing efficiency when handling massive point cloud data. To improve computational efficiency, researchers have implemented voxelization methods to reduce point cloud quantities [50]. However, applying uniform voxel grids in dense rubber forest areas may result in the loss of authentic tree shape information. The Density-Based Spatial Clustering of Applications with Noise (DBSCAN) algorithm [51] is a density-based point cloud clustering method that effectively eliminates noise interference and enables clustering into arbitrary shapes, thus preserving the true morphology of trees.

In this study, the rubber plantation point cloud is segmented at height intervals of *ΔHL* = 0.2 m to ensure the complete clustering of point clouds within each layer for obtaining skeleton points. Maintaining sufficient points in each cluster effectively prevents local data loss. The DBSCAN algorithm clusters the point cloud of each layer, and the centroid of each cluster is calculated. These centroids serve as the initial skeleton points of rubber trees across the forest area, as expressed in Equation (1).

(1) $$c_{i,j}=\frac{1}{N_{i,j}}\sum_{k=1}^{N_{i,j}}\left(x_{i,j,k},y_{i,j,k},z_{i,j,k}\right)$$

where $c_{i,j}$ represents the centroid (skeleton point) of cluster *j* in layer *i*, $N_{i,j}$ represents the number of points in cluster *j* of layer *i*, and $x_{i,j,k}$, $y_{i,j,k}$, and $z_{i,j,k}$ respectively represent the coordinates of point *k* in cluster *j* of layer *i*.

#### 4.3.2. Extraction of the Skeleton of Individual Tree Main Trunks

Due to the density disparities between the trunk and canopy, it is challenging to apply a uniform density threshold for segmenting both simultaneously. Therefore, accurately identifying the branch points between the trunk and canopy, while adopting distinct density thresholds for each, is crucial for the extraction process. To address this, we utilize two constraints, neighborhood point density and the main direction [52], to define the thresholds for neighborhood point density and upward search angle.

**Figure 7:**
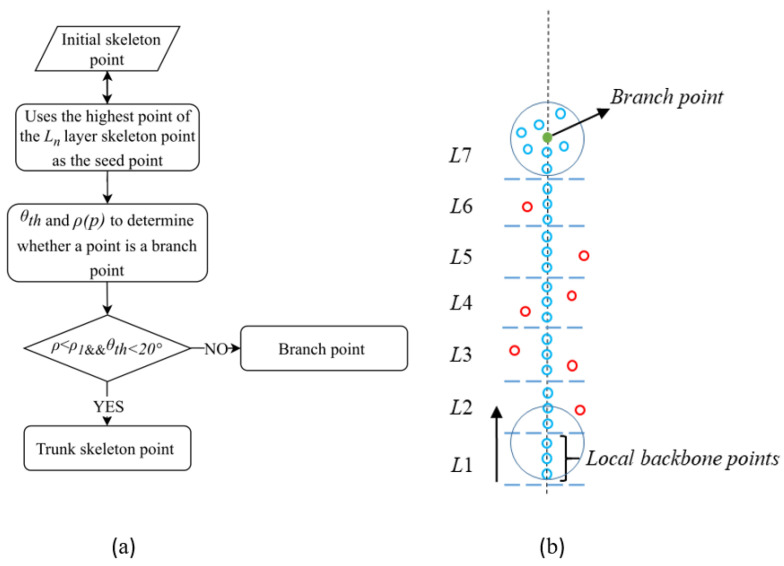
Extraction of individual tree trunk skeleton points. (**a**) Trunk skeleton point extraction process; (**b**) schematic diagram of trunk skeleton point extraction. In layer L1, skeleton points serve as the local backbone. Moving from bottom to top, skeleton points (blue dots) meeting the specified criteria are identified as trunk components. Red dots indicate abnormal points that either deviate from the local backbone’s primary direction or are identified as noise and subsequently removed. Trunk growth terminates when the number of neighboring points exceeds the threshold *ρ*_1_, signifying the presence of a branch point (green dots).

The process begins by taking the lowest elevation point of the local trunk as the seed point, followed by an upward search within a specified radius neighborhood. Skeleton points that are smaller than the neighborhood point density threshold and comply with the angle threshold are merged into the trunk skeleton points. Considering the straight nature of rubber tree trunks and the increased canopy point density at branch points, the process continues until the branch point is identified. All skeleton points beneath the branch point are then classified as part of the trunk, thus completing the trunk segmentation. A schematic of this process is shown in Figure 7.

(1) Determine the seed point: The local trunk Ln is obtained through DBSCAN, with the highest elevation point of the local trunk skeleton serving as the seed point to search for neighboring points. The density threshold, *ρ*_1_, is set to identify the branch point. If the number of unclassified skeleton points within the search range is less than *ρ*_1_, the current point is classified as a local trunk skeleton point.

(2) Trunk skeleton growth rules and eigenvalue analysis: Eigenvalues and their corresponding eigenvectors are estimated by calculating the covariance matrix *M* of the points within the neighborhood [34]. The covariance matrix *M* of the skeleton points in the local trunk cluster $S=\{s_{i}|i=1,2,\cdots ,N_{s}\}$ and the *q* unclassified skeleton points is presented in Equation (2).

(2) $$M=\frac{1}{N+Q}\sum_{i=1}^{N+Q}\left(s_{i}-\bar{S}\right)(s_{i}-\bar{S}{)}^{T}$$

In the equation, *N* denotes the number of skeleton points in the local main trunk cluster, *Q* represents the number of skeleton points to be classified, and $\bar{S}$ is the center of the local main trunk skeleton and the skeleton points to be classified. The eigenvector corresponding to the maximum eigenvalue defines the main direction. Due to environmental perturbations, the rubber tree’s main trunk may not be perfectly perpendicular to the ground. Based on experiments and experience, the angle tolerance *θ*th is set to 20°. If the angle between the skeleton point to be classified and the main direction of the main trunk is less than *θ*th and the neighboring point density is smaller than *ρ*_1_, the point is added to the main trunk skeleton points *S*. This rule is outlined in Equation (3).

(3) $$G=\left\{\begin{array}{ll} L_{i=0}^{n}\{P\in S_{i}|f\left(\cos^{-1}(S_{x}V_{x}+S_{y}V_{y}+S_{z}V_{z})\cdot \frac{180^{{}^{\circ}}}{\pi }\right)<20^{{}^{\circ}}\wedge \rho (P)<\rho_{1}\}, & Trunk\;skeleton\;point,\\ L_{i=0}^{n}\{P\in S_{i}|f\left(\cos^{-1}(S_{x}V_{x}+S_{y}V_{y}+S_{z}V_{z})\cdot \frac{180^{{}^{\circ}}}{\pi }\right)>20^{{}^{\circ}}\wedge \rho (P)>\rho_{1}\}, & Branch\;point. \end{array}\right.$$

In the equation, $S(S_{x},S_{y},S_{z})$ represents the normalized vector from the skeleton point to the classified point, and $V(V_{x},V_{y},V_{z})$ is the normalized vector of the main direction. The function *f* determines whether the point is a branch point based on angle and density, and *ρ(P)* is the neighborhood density of the seed point.

(3) Trunk skeleton extraction based on density and principal direction characteristics: The highest elevation point from *S* is selected as the new seed point, and Steps 1 and 2 are repeated until the number of neighboring unclassified skeleton points exceeds the threshold *ρ*_1_ and falls outside the angle tolerance *θ*th, signaling that the branch point has been reached. This marks the conclusion of the trunk skeleton extraction process.

#### 4.3.3. Multi-Feature Fusion-Based Extraction of the Canopy

Rubber forests typically feature a high canopy closure, making it difficult to effectively segment individual trees with heavily overlapping branches using a single feature. To address this challenge, this paper introduces an Integrated Tree Skeleton (ITS) strategy that combines multiple features, including distance, local density, and angle. The proposed strategy defines distinct segmentation methods for skeleton points at various positions, such as core points, boundary points of the tree crown, and disputed points where branches overlap.

First, the distance characteristic from the skeleton points of the tree crown to the branch points is defined. This, combined with the local density feature of neighborhood growth, allows for the sequential segmentation of the core and boundary points of the tree crown. Next, a discrimination method based on the consistency of the growth direction angle of the attributed points within the neighborhood is introduced to determine the attribution of disputed points in areas of branch overlap at the canopy boundary.

**Figure 8:**
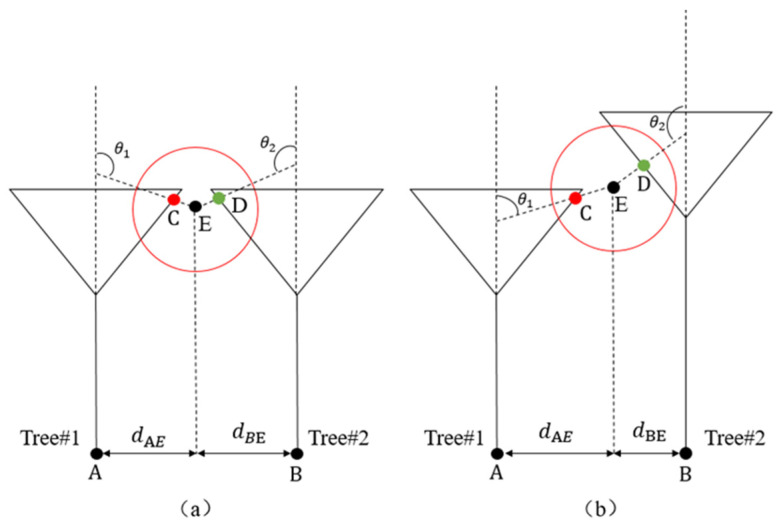
Determining the ownership of disputed points using an angle threshold. (**a**) Homogeneous Competition: When Tree#1 and Tree#2 have similar growth directions, the angle θ_1_ between the direction vector from E to C and the main growth direction of Tree#1 is compared with the angle *θ*_2_ between the direction vector from E to D and the main growth direction of Tree#2. If *θ*_1_ < *θ*_2_, E is assigned to Tree#1. The angle threshold (e.g., 60°) filter mismatches. (**b**) Heterogeneous Competition: When Tree#1 and Tree#2 have divergent growth directions, θ_1_ is significantly smaller than θ_2_, making E more likely to be assigned to Tree#1. This ensures accurate segmentation under varying canopy structures.

For example, taking branch points A and B as the centers of the tree crowns, as illustrated in Figure 8, skeleton points within a horizontal distance less than *T_HD1_* from the branch points and with a local density *ρ* ≥ *ρ*_3_ are assigned to the tree corresponding to the branch point. These points are classified as core skeleton points of the tree crown, as described in Equation (4). Skeleton points within a horizontal distance range of (*T_HD1_*, *T_HD2_*) from the branch points, with a local density *ρ* < *ρ*_3_, are considered boundary skeleton points of the tree crown. A nearest distance search is then conducted on the boundary points, which are assigned to the tree of the nearest segmented point, as shown in Equation (5). Points within the horizontal distance range (*T_HD1_*, *T_HD2_*) and with a local density *ρ* > *ρ*_2_ are regarded as disputed points in areas with significant branch overlap, as represented in Equation (6).

Typically, the branches of rubber trees grow at an upward angle from the main trunk. Based on this characteristic, a neighborhood search is conducted from the disputed point E, and the skeleton points C(*C_x_*, *C_y_*, *C_z_*) and D(*D_x_*, *D_y_*, *D_z_*) with the smallest distance are selected. *V_EC_* and *V_DE_* are the vectors from point E to points C and point D, respectively. *θ*_1_ is the angle between *V_EC_* and the main direction of Tree #1, while *θ*_2_ is the angle between *V_ED_* and the main direction of Tree #2. If *θ*_1_ is smaller than *θ*_2_, point E is assigned to Tree #1 (Figure 8a); conversely, E is assigned to Tree #2 (Figure 8b).

(4) $$H\in Core\;point\;of\;tree\;crown,\;\mathrm{if}\;d_{HA}\in \left(0,\;T_{HD1}\right)\&\rho \geq \rho_{3}$$

(5) $$H\in Boundary\;points,\;\mathrm{if}\;d_{HA}\in \left(T_{HD1},T_{HD2}\right)\&\rho <\rho_{3}$$

(6) $$H\in Disputed\;points,\;\mathrm{if}\;d_{HA}\in \left(T_{HD1},T_{HD2}\right)\&\rho <\rho_{2}$$

where *H* denotes the skeleton point classification attribute, *T_HD1_* represents the minimum crown radius, *T_HD2_* represents the maximum crown radius, and *ρ*_2_ and *ρ*_3_ represent the local densities of skeleton points within the radius range.

Since disputed points predominantly occur in areas of severe branch overlap and typically have density values lower than *ρ*_3_, *ρ*_2_ should be set lower than *ρ*_3_. Based on the average branching angles in the sample plots, angle thresholds are set at 60° for plots A and B, and 50° for plot C. In cases where skeleton points are missing within the neighborhood range, disputed points are assigned based on the nearest distance principle. The process of the Multi-Feature Fusion Canopy Extraction is shown in Algorithm 1:
**Algorithm 1** Multi-Feature Fusion Canopy ExtractionInput: Skeleton points *S*, Branch points *B*, Thresholds: *T_HD1_*, *T_HD2_*, *ρ*_2_, *ρ*_3_, *θ_th_* Output: Classified labels *L*(*s*) ∈ {*TreeID*, unclassified} 1:  Initialize all *s* ∈ *S* with *L*(*s*) = unclassified 2:  Core Crown Point Identification for each branch point *b*_*j*∈*B* do 3:  *S*_candidates = {*s* ∈ *S* | dist(s, *b*_*j*) ≤ *T_HD1_*} 4:   for each point *s*∈*S*_candidate do 5:   if *ρ*(*s*) ≥ *ρ*_3_ then 6:   *l*(*s*) ← *j* // Mark as core point of tree *j* 7:   end if 8:   end for 9:  end for 10:   Boundary and Disputed Point Classification for each unclassified point *s*∈*S* do 11: Compute distance to nearest branch point *d* = min(dist(*s*, *b*_*j*)) 13:  if *T_HD1_* < *d* ≤ *T_HD2_* then 14:   if *ρ*(*s*) < *ρ*_2_ then 15:    *l*(*s*) ← *TreeID* of nearest classified point // Boundary point 16:   else if *ρ*(s) < *ρ*_3_ then 17:    *l*(*s*) ← resolve_directional_conflict(*s*) // Handle disputed point 18:    else 19:    l(s) ← treeID of nearest branch point // High-density peripheral point 20:   end if 21:  end if 22:   end for

#### 4.3.4. Accuracy Assessment of ITS

To evaluate the accuracy of the ITS algorithm presented in this paper, the crown contours of each individual tree in each sample plot are verified on-site, and the point clouds of individual trees are manually segmented as a reference. The ratio of the number of points segmented by the algorithm to those manually segmented (*ρ_algorithm_*_/reference_) is used to assess segmentation accuracy (see Table 3). The recall (*R*), precision (*P*), and harmonic mean (*F*) are utilized to evaluate the performance of individual tree detection (Equations (7)–(9)). Recall (*R*) indicates the ratio of accurately identified rubber trees to the total number of rubber trees, while precision (*P*) represents the proportion of correctly detected trees in the total detection outcomes. The ITS algorithm classifies the point cloud into multiple categories, where true positive (*TP*) represents the number of correctly segmented trees; false negative (*FN*) represents the number of trees not segmented but assigned to a nearby tree (under-segmentation); and false positive (*FP*) represents the number of trees that did not exist but were segmented from the point cloud (commission error or over-segmentation).

(7) $$R=\frac{TP}{TP+FN}$$

(8) $$P=\frac{TP}{TP+FP}$$

(9) $$F=2\times \frac{R\times P}{R+P}$$

The canopy projection area refers to the area encompassed by the concave polygon formed by the boundary points of an individual tree canopy [53,54]. The crown width is defined as the mean of the crown widths measured in both the east–west and north–south orientations. The coefficient of determination (*R*^2^), root mean square error (*RMSE*), and normalized root mean square error (*NRMSE*) are used to evaluate the accuracy of parameter extraction in this paper, thereby verifying the accuracy of ITS. A value of *R*^2^ close to 1 and a smaller *RMSE* suggest a high extraction accuracy, while a smaller *NRMSE* indicates better consistency between the extracted values and the measured values.

(10) $$R^{2}=1-\frac{\sum_{i=1}^{n}{\left(y_{i}-\hat{y}\right)}^{2}}{\sum_{i=1}^{n}\left(y_{i}-\bar{y}\right)}$$

(11) $$RMSE=\sqrt{\frac{1}{n}\sum_{i=1}^{n}{\left(y_{i}-{\hat{y}}_{i}\right)}^{2}}$$

(12) $$NRMSE=\frac{RMSE}{\bar{y}}\times 100\%$$

where $y_{i}$ represents the measured value of the individual tree structural parameters; ${\hat{y}}_{i}$ represents the value of the individual tree structural parameters extracted by the algorithm; $\bar{y}$ represents the average measured value of the individual tree structural parameters; and *n* represents the number of rubber tree samples.

## 5. Conclusions

In addressing the challenges of ITS in complex rubber plantations characterized by indistinct canopies and branch overlap, this paper presents a novel bottom-up growing ITS method utilizing LiDAR point clouds. The method innovatively combines main trunk extraction based on branch point identification with a multi-feature fusion approach, effectively segmenting individual trees across varying canopy closure levels with high accuracy (0.97, 0.98, and 0.95 for low, medium, and high closure plots, respectively), outperforming existing algorithms such as the hierarchical DBSCAN, ForAINet, PCS, layer stacking, K-means, and watershed. Validated by strong R^2^ values for crown morphology parameters, the proposed approach provides a robust and reliable solution for precise ITS, offering a crucial foundation for accurate structural parameter extraction, three-dimensional model construction, and biomass inversion in rubber plantations. Future research directions include mitigating understory vegetation influence, developing adaptive thresholding, and exploring soft segmentation and multi-sensor data fusion to further enhance segmentation accuracy and automation, ultimately contributing to the precise management and sustainable development of rubber plantation ecosystems.

## Figures and Tables

**Table 1:** Accuracy evaluation of single-wood segmentation comparative experiment.

Study Area	Method	Ground Truth of Total Tree Count	*TP*	*FN*	*FP*	*R*	*P*	*F*
Plot A	Proposed approach	38	36	1	1	0.97	0.97	0.97
	DBSCAN	38	31	5	2	0.86	0.94	0.90
	PCS	38	28	7	3	0.80	0.90	0.85
	Layer stacking	38	23	8	7	0.74	0.77	0.75
	K-means	38	18	10	10	0.64	0.64	0.64
	ForAINet	38	13	10	15	0.56	0.46	0.55
	Watershed	38	11	17	10	0.39	0.52	0.45
Plot B	Proposed approach	32	31	1	0	0.97	1.00	0.98
	DBSCAN	32	22	5	5	0.81	0.81	0.81
	PCS	32	19	7	6	0.73	0.76	0.75
	ForAINet	32	18	2	12	0.90	0.60	0.72
	Layer stacking	32	10	12	10	0.45	0.50	0.48
	K-means	32	8	13	11	0.38	0.42	0.40
	Watershed	32	2	18	12	0.10	0.14	0.12
Plot C	Proposed approach	32	29	2	1	0.94	0.97	0.95
	DBSCAN	32	15	10	7	0.60	0.68	0.64
	ForAINet	32	7	10	15	0.41	0.32	0.36
	PCS	32	6	14	12	0.30	0.33	0.32
	Layer stacking	32	5	16	11	0.24	0.31	0.27
	K-means	32	3	18	11	0.14	0.21	0.17
	Watershed	32	1	22	9	0.04	0.10	0.06

**Table 2:** The basic information of the three sample plots.

Study Area	Area (m^2^)	Canopy Closure Level	Canopy Closure (%)	Total Tree Number	LiDAR Point Density (pt/m^2^)	Total LiDAR Points (×10,000)
Plot A	1189.9	Low	41.5	38	2114	70.8
Plot B	376.323	Medium	68.9	32	1384	46.6
Plot C	404.212	High	89.3	32	3110	97.3

**Table 3:** Accuracy assessment criteria based on the percentage of points.

Criteria of the Percentage of Points	Segmentation Category
*ρ*_algorithm/reference_ > 105%	Correctly segmented
105% ≥ *ρ*_algorithm/reference_ ≥ 95%	Over-segmented
*ρ*_algorithm/reference_ < 95%	Under-segmented

## Data Availability

Data are contained within the article. Further inquiries can be directed to the corresponding authors.
